# Acute hemorheological responses to an incremental shuttle-run test at -10 °C versus 18 °C in young healthy men

**DOI:** 10.3389/fphys.2026.1935076

**Published:** 2026-10-14

**Authors:** Aneta Teległów, Konrad Rembiasz, Janusz Pobędza, Łukasz Cebula, Zygmunt Dziechciowski, Andrzej Czerwiński, Jakub Leśniowski, Jakub Marchewka, Piotr Mika

**Affiliations:** 1 Department of Health Promotion, Faculty of Motor Rehabilitation, University of Physical Culture, Krakow, Poland; 2 Indoor Swimming Pool Complex, University of Physical Culture, Krakow, Poland; 3 Laboratory for Climate Technology Research and Heavy Duty Machines, Faculty of Mechanical Engineering, Cracow University of Technology, Krakow, Poland; 4 Faculty of Motor Rehabilitation, University of Physical Culture, Krakow, Poland; 5 Department of Rehabilitation in Traumatology, Faculty of Motor Rehabilitation, University of Physical Culture, Krakow, Poland; 6 Institute of Clinical Rehabilitation, Faculty of Motor Rehabilitation, University of Physical Culture, Krakow, Poland

**Keywords:** blood plasma viscosity, fibrinogen, low temperature, shuttle run test, thermal climate chamber

## Abstract

**Background:**

The presented study concerns exercise physiology under conditions of effect of single sprint in air temperature of –10 °C on selected blood hemorheological indicators (fibrinogen, blood plasma viscosity) in healthy males. Important to know the differences in hemorheological indicators on physical exercise at low temperatures compared to those obtained at normal temperatures cardiovascular risk assessment.

**Methods:**

The study was experimental in nature and assumed a comparison of the body response to exercise performed at reduced (–10 °C) and neutral (18 °C) temperatures. It involved 30 healthy males, students of physiotherapy at the University of Physical Culture in Krakow, Poland. The enrolled individuals were divided into 2 groups: group 1 (*n* = 15) ran in a thermal climate chamber at an air temperature of –10 °C, group 2 (*n* = 15) ran in the fresh air at a neutral air temperature of 18 °C.

**Results:**

Significant changes were observed in the platelet count in both the control group and the experimental group. No effect of the experimental factor, i.e. cold, was noted for the following indicators: fibrinogen, blood plasma viscosity, platelet indicators.

**Conclusions:**

Prevailing temperature conditions during single sprint had no substantial influence on changes in the analyzed blood parameters. The main finding of the study is that short-term, maximal single sprint in very low temperatures (-10 °C) does not induce different or more pronounced hemorheological changes than the same effort performed under thermoneutral conditions (18 °C) in young, healthy males. The results suggest that a short, intense workout in temperatures of -10 °C, when wearing appropriate clothing (thermal base layers), does not lead to dangerous disturbances in blood flow.

## 1 Introduction

A single bout of physical activity primarily affects the cardiorespiratory system, which must adapt to the new conditions, but intensive, acute exercise may sometimes constitute a risk of acute cardiovascular accidents (Toth et al., 2007). Exposure to cold—which triggers peripheral vasoconstriction, raises arterial blood pressure, and consequently increases the workload on the heart—is a significant factor; if this were accompanied by a substantial increase in plasma viscosity and the release of fibrinogen, a state of hypercoagulability would ensue (Keatinge et al., 1984). Our research indicates that, in healthy young individuals, compensatory mechanisms prevent plasma hemoconcentration and the increased risk of thrombosis during short-term exercise in cold temperatures. This explains why cardiovascular incidents during physical activity in the cold (e.g., winter sports) primarily affect older adults or those with coronary artery disease, in whom adaptive mechanisms are impaired (Gao et al., 2012). According to Brun et al (Brun, 2002), the impact of physical exertion on the rheological properties of blood is triphasic in nature, comprising short-term effects—namely increased blood viscosity resulting primarily from body fluid shifts and changes in erythrocyte rigidity and aggregability—followed by medium-term effects (so-called autohemodilution), which lead to a reduction in both plasma viscosity and hematocrit levels, and finally long-term effects that further improve blood fluidity, coinciding with classic training-induced hormonal and metabolic changes. These processes are also accompanied by changes in leukocyte counts and oxidative stress (Brun, 2002).

It is estimated that during maximum exertion, total blood flow increases 5- to 8-fold as compared with resting conditions, with as much as 84–85% of that flow passing through the working skeletal muscles. Blood flow through the brain remains unchanged, while that through the viscera and kidneys decreases; cutaneous blood flow depends on other factors, such as body temperature and ambient temperature. During physical effort, 75% of the energy produced is converted into heat, which triggers mechanisms aiming to maintain a relatively constant body temperature: sweating and sweat evaporation (Teległów, 2024). During intense physical activity, the body internal temperature can reach 39 °C (Sawka et al., 2012). The greater the difference between core body temperature and skin temperature, the more effectively the body dissipates excess heat and prevents hyperthermia: reduced blood flow through the skin releases the same amount of heat, which preserves cardiac efficiency without forcing increased heart rate (Teległów, 2024).

A single bout of physical effort also affects changes in the parameters of circulating blood (Bilski et al., 2014). In the short term, it leads to a temporary increase in blood viscosity, mainly because of raised hematocrit and plasma viscosity (increased protein concentration), but also owing to changes in red blood cells (decreased deformability and increased aggregation) (Martin et al., 1985; Brun et al., 2010; Bilski et al., 2014). It is assumed that this is caused by oxidative stress and increased levels of lactic acid. Regular physical exertion results in an opposite adaptive change: a decrease in hematocrit and an improvement in erythrocyte rheology (including increased deformability and reduced aggregation) (Brun et al., 2010). After a single bout of maximum physical exertion, the plasma fibrinogen concentration decreases, only to return to normal after half an hour of rest (El-Sayed et al., 1999). This is believed to result from increased circulating blood volume during exercise (owing to, among others, spleen contraction) and, as a consequence, from elevated plasma volume (Teległów, 2024).

In their meta-analysis, Heber and Volf (2015) concluded that strenuous, intense physical effort could lead to platelet activation, i.e., an increase in their number and activity in the bloodstream. In a study involving 31 half-marathon runners, Lippi et al. (2014) demonstrated an increase in platelet count and a slight increase in mean platelet volume immediately after the race. After approximately 24 hours, the indicators returned to normal. The authors were unable to explain why the platelet count had increased (Rajzer et al., 2007). This can be attributed mainly to their release from the spleen and lungs. The neutral ambient temperature for a naked human being is 27 °C. Exposure to cold stimulates skin receptors, which activates the sympathetic nervous system, leading to skin blood vessel constriction and blood flow centralization. Vasoconstriction induced by cold increases blood pressure, which in turn translates into a greater strain on the heart. A rise in blood pressure can be counteracted by light exercise, but intense exercise exacerbates the blood pressure rise (Rintamäki, 2007). As a reaction to an increase in blood pressure, the heart rate slows down (Teległów et al., 2024). With exposure to cold, vasoconstriction increases blood viscosity and reduces plasma volume, which may affect the risk of cardiovascular events (Neild et al., 1994; Rintamäki, 2007; Ni et al., 2022). Cold exposure also causes an increase in platelet count (Lombardi et al., 2011; Lippi et al., 2014). Blood viscosity measurements taken at a temperature range of 22–45 °C in previously collected samples showed that viscosity increased with decreasing temperature, but the change was not linear across the entire temperature range. The most significant change was observed at lower temperatures (Urbanová, 1996; Cinar et al., 2001). The impact of short-term exposure to cold on fibrinogen concentration remains unclear. Some studies report a significant increase in its levels, particularly among older adults (Neild et al., 1994); in turn, other research suggests no change in its concentration (Teległów et al., 2024). It is believed that a potential increase in fibrinogen levels may be partly related to an inflammatory response (Neild et al., 1994).

The purpose of this study was to assess selected blood hemorheological indicators (fibrinogen, blood plasma viscosity) as affected by single sprint at low temperature (–10 °C) in healthy males.

## 2 Materials and methods

### 2.1 Study group characteristics

The study involved 30 healthy males, students of the University of Physical Culture in Krakow, Poland, faculty of physiotherapy. Their mean body height was 178.9 ± 6.5 cm, mean body mass equaled 76.8 ± 11.3 kg. After the subjects were assigned to each group, the average body height in the experimental group (177.9 ± 5.9 cm) and in the control/neutral group (179.9 ± 7.2 cm) was calculated. The average body mass in the experimental group (73.1 ± 6.6 kg) was lower than that in the control group (81.2 ± 14.1 kg). The inclusion criteria for the study were as follows: subjects were required to be aged 22 years, male, not to have undergone competitive training in any sport, and free of chronic diseases or contraindications to physical activity, Non-smoker, declared alcohol abstinence. Participants were excluded from the study if they met any of the following criteria: age above or below 22 years, chronic cardiovascular or respiratory diseases, hematological disorder, eating disorders, symptoms of infection (fever, cough, rhinitis), or competitive participation in any sport. The participants were also informed in detail about the purpose and procedure of the study, as well as the fact that they could withdraw from participation at any time without any consequences. The choice of a narrow age group (22 years old) and a single gender was intended to minimize the effect of confounding variables to the greatest extent possible, including age, life and work experience, as well as gender-related hormonal and psychological differences.

Simple randomization with a 1:1 allocation ratio served to randomly assign the participants to the experimental or control group. This was done before the first blood sampling, with the randomization sequence generated prior to the study. No stratification factors were used. The participants in the experimental group performed physical exertion under the conditions of low temperature (–10 °C). The workout was undertaken in a thermal climate chamber in the Laboratory for Climate Technology Research and Heavy Duty Machines, Cracow University of Technology in Krakow. The thermal climate chamber meets the requirements of the PN-EN ISO/IEC 17025:2005 standard and possesses an accreditation certificate No. AB 1678, issued by the Polish Centre for Accreditation. The Laboratory for Climate Technology Research and Heavy Duty Machines is a research center accredited in the field of defense and security by the Minister of National Defense (accreditation No.: 55/MON/2018). The control group, in turn, performed the same physical effort but in the open air, in thermoneutral conditions (the air temperature was 18.1 °C on the day of the study).

The experiment comprised 3 stages. Blood samples were collected from fasting subjects:

1. Before the exercise, blood samples were taken from the participants in the morning hours by a qualified nurse, The blood was taken from each participant’s anterior ulnar vein into Vacuette EDTA K2 tubes. The subjects drank 500 ml of water on an empty stomach before a blood sample was drawn from the ulnar vein. This was performed in the morning hours on 11 May 2024 at the Blood Physiology Laboratory of the Central Research and Development Laboratory, University of Physical Culture in Krakow.
2. Both groups undertook an exertion test.
3. Blood samples were collected from all participants again after the exercise. Blood samples were collected from fasting subjects. The nurse drew blood immediately after the men exercised.

The study was approved by the Ethics Committee of the Regional Medical Chamber in Krakow, Poland (approval No.: 97/KBL/OIL/2024) and followed the tenets of the Declaration of Helsinki.

### 2.2 Research method

A two-factor experimental design with repeated measures was employed in the study. Ambient temperature (low vs. thermoneutral) constituted the between-subject factor; time of measurement (pre- and post-exercise) was the within-subject factor. The study followed the design of an exploratory investigation. In the group allocation of the participants, the principle of double randomization was employed. In experimental research, where a phenomenon is induced by the researcher on purpose, its course is observed in a planned and controlled way. Therefore, the influence of particular factors on the studied parameters can be assessed. Conclusions are drawn on the basis of the discovered relationships; the accuracy of the conclusions is evaluated with a specific probability. In the present study, a significance level of α = 0.05 was assumed, corresponding to a 95% confidence level.

### 2.3 Parallel group technique

The participants were randomly allocated to the experimental group and the control group; the double randomization principle was followed. The first measurement preceded introducing the experimental factor.

The two groups were observed but only the experimental group received the additional stimulus, namely low temperature, i.e. the experimental factor. A second measurement was then performed, allowing to evaluate the changes imposed by the intervention. Owing to the application of a control group, it was possible to state if the reported changes resulted from the experimental factor – and not from other variables, which were uncontrolled. Although confounding variables are not regarded as essential in the main analysis, they can impact on the research results, leading to effect misinterpretation. Here, exposure to low ambient temperature constituted the experimental factor. The testing environments were different in the two groups (controlled thermal climate chamber in the experimental group and natural outdoor conditions in the control group), which was taken into consideration when interpreting the results.

### 2.4 Shuttle run test

In the current study, cardiorespiratory capacity was determined with the use of an endurance shuttle run test. This is a standardized, incremental protocol commonly applied to provoke progressively increasing cardiorespiratory load. Direct physiological intensity indicators were not recorded but the same protocol was used in both groups in order to obtain comparable external workload progression.

The test started with a steady pace, which equaled 8 km/h. Subsequently, the running speed increased by 0.5 km/h per minute. The subjects ran a 20-m distance back and forth and then touched the finish line with one foot. The running pace was indicated by a beep. The participant finalized the test if they failed to reach the line at a distance of at least 3 m before the beep sound for two successive running sections. The number of completed sections was considered the test result (Toth et al., 2007) (Léger and Gadoury, 1989).The subjects then took part in an endurance shuttle run conducted until exhaustion under the supervision of a trainer (**Table 1**).

**Table 1:** Results for the experimental group and the control group for duration and VO_2_Max.

	Study group		Control group	
	W	p	W	p
DURATION	0.946	0.463	0.909	0.152
VO_2_max	0.943	0.419	0.939	0.412

**Table 1** presents the results of the normality distribution tests for variables related to physical exercise. The analysis showed that the distribution of values for duration of exercise and estimated maximal oxygen uptake (VO_2max_) in both study groups did not deviate from normality.

### 2.5 Methods of blood analysis

A qualified nurse collected fasting blood samples in 10-ml Vacuette tubes in the morning hours. Blood samples were collected from fasting subjects. The blood was taken from each participant’s anterior ulnar vein into Vacuette EDTA K2 tubes. The staff at the Blood Physiology Laboratory of the Central Research and Development Laboratory, University of Physical Culture in Krakow supervised the procedure, following the applicable standards.

### 2.6 Morphological assessments

The following blood count parameters were assessed: platelet count (PLT) [10^3^/mm^3^], mean platelet volume (MPV) [μm^3^], platelet distribution width (PDW) [%]. The measurements were performed by using an ABX Micros 60 hematology analyzer (Horiba ABX SAS, Montpellier, France).

### 2.7 Fibrinogen

Fibrinogen concentration [g/l] was determined with the use of a CHROM-7 coagulometer (Poland).

### 2.8 Blood plasma viscosity

Plasma viscosity (PV) [mPa·s] was assessed with a DVNext Brookfield Ametek Cone/Plate rheometer (Brookfield, Middleboro, MA, USA). The device uses a calibrated beryllium-copper spring to measure torque generated by the resistance of the plasma sample between a rotating cone and a stationary plate. The measurements were conducted at 37 °C with a CP-40Z spindle at shear rate of 450 s^–1^.

### 2.9 Statistical analysis

The differences between pre- and post-exercise measurements in the experimental and control groups were determined by using a two-way analysis of variance with repeated measures and Bonferroni’s multiple comparison test. The distribution of the variables was assessed with the Shapiro-Wilk test, the homogeneity of variances with Levene’s test, and the sphericity of variances with Mauchly’s test. A 95% confidence level was assumed in the data analysis. Test probability values of 0.05 or less were considered statistically significant. Values less than 0.01 indicated high statistical significance, and values less than 0.001 implied extremely high statistical significance. The calculations were performed with the use of the Statistica 13.1 software (StatSoft Polska).

## 3 Results

No statistically significant differences were observed after the physical effort in either of the study groups for the following parameters: PDW, MPV, platelet-large cell ratio (P-LCR), PV, fibrinogen (*p* > 0.005). The only statistically significant differences were reported for PLT in both groups: the experimental one (*p* < 0.001) and the control one (*p* < 0.001), as well as for plateletcrit (PCT, platelet hematocrit) in the experimental group (*p* = 0.017) (**Table 2**).

**Table 2:** Results for the experimental group and the control group before and after the effort.

	Experimental group						*p*	Control group						*p*	*p* (E–C)
	Before			After				Before			After				
	Mean	±	SD	Mean	±	SD		Mean	±	SD	Mean	±	SD		
PLT [10^3^/mm^3^]	244.36	±	33.10	302.47	±	49.49	< 0.001*	227.14	±	53.95	263.21	±	61.41	< 0.001*	0.230
PDW [fl]	13.00	±	2.09	12.88	±	1.99	0.810	12.52	±	2.05	12.92	±	2.08	0.883	1.000
MPV [f/L]	10.66	±	0.94	10.61	±	0.96	0.773	10.54	±	0.88	10.69	±	1.08	0.993	1.000
P-LCR [%]	30.76	±	7.90	30.21	±	7.95	0.700	29.16	±	7.26	31.04	±	8.86	0.806	1.000
PCT [%]	0.27	±	0.05	0.31	±	0.05	0.017*	0.25	±	0.07	0.28	±	0.06	0.143	0.334
BPV [mPa·s]	1.50	±	0.06	1.55	±	0.06	0.144	1.52	±	0.05	1.57	±	0.09	0.063	0.763
Fibrinogen [g/L]	3.02	±	0.40	3.11	±	0.44	0.919	3.16	±	0.47	3.02	±	0.53	0.699	0.994

PLT, platelet count in a given volume; PDW, platelet distribution width, marker of anisocytosis; MPV, mean platelet volume; P-LCR, platelet-large cell ratio, percentage of platelets deviating from normal; PCT, plateletcrit, platelet hematocrit; BPV, blood plasma viscosity;.

*p* (E–C), level of statistical significance of between-group differences experimental and control.

*statistical significance (*p* ≤ 0.05).

No statistically significant differences in indicator changes were found between the experimental group and the control group. Detailed results for the experimental group, the control group, and the statistical significance of the between-group differences are presented in **Table 2**.

## 4 Discussion

The presented study demonstrated the impact of single sprint an increase in platelet count, in young men in blood collected immediately post-exercise. These findings confirm those obtained by Heber and Volf (2015) and by Lombardi et al. (2011). It is believed that this change is primarily caused by the release of platelets from the spleen and lungs (Teległów, 2024). However, there were no statistically significant differences between the groups: most likely, the effect of low temperature did not exacerbate the described phenomenon. The experimental group also showed an increase in platelet hematocrit in air temperature of –10 °C, which could suggest that exposure to cold led to a decrease in plasma volume (Neild et al., 1994; Lombardi et al., 2011; Ni et al., 2022). However, no statistically significant differences were reported between the experimental and control groups; it must therefore be assumed that these changes were not caused by the low temperature, but by other factors (e.g., group-specific characteristics for young men). No changes were observed in the other platelet parameters.

Blood plasma viscosity did not change significantly in either group in young men in blood collected immediately post-exercise (single sprint), which is somewhat surprising in light of the cited scientific reports which clearly demonstrated an increase in viscosity both during exercise (Martin et al., 1985; Brun et al., 2010; Bilski et al., 2014; Teległów, 2024) and upon exposure to cold (Neild et al., 1994; Rintamäki, 2007; Ni et al., 2022). It was expected that these two effects would overlap, resulting in a significant increase in this parameter in the experimental group and an increase in the control group. Furthermore, the effect of low temperature on viscosity appears to be independent of the duration and intensity of cold stimulation, as demonstrated by Keatinge et al. (1984). These authors subjected eight students to prolonged, six-hour stimulation with moving air at a temperature of 24 °C. They observed increased blood viscosity and platelet count and considered this a normal thermoregulatory adaptation (Keatinge et al., 1984).

One should, however, note that most studies examine the viscosity of whole blood, which includes non-plasma factors, such as erythrocyte properties and hematocrit. This leads to a conclusion that the changes in whole blood viscosity observed in the aforementioned studies are primarily a result of changes in erythrocyte elongation and aggregation, as well as in hematocrit, rather than changes in the plasma itself or its proteins. On the other hand, a study by Letcher et al. (1981), conducted among both trained runners and sedentary individuals, suggests an increase in plasma viscosity in both groups after intense physical exertion on a treadmill in accordance with the Bruce protocol. The authors emphasize that this increase was not as high as expected. Alis et al. (2015), when examining the impact of high-intensity interval training compared with continuous running, observed increased plasma viscosity only after the interval effort. This study may suggest that the greater the exercise intensity, the larger the change in plasma viscosity. However, this does not translate into the own study results, in which a maximum-intensity protocol conducted until exhaustion was applied. Brun et al. (2007) state that physical exertion often causes an increase in plasma viscosity as a result of the body’s loss of water. In the own study, the low ambient temperature likely did not force the participants to sweat as intensely as exercise in the open air to maintain a constant core temperature, which – in accordance with the assumptions by Brun et al. (2007) – may not have resulted in increased viscosity. On the other hand, in young men in blood collected immediately post-exercise (single sprint) one would then expect raised viscosity in the control group, who exercised outdoors at a temperature of 18 °C; however, no such changes were observed. The issue of changes in plasma viscosity due to physical exertion at low temperatures therefore remains unclear, and further research is needed in this area in young men in blood collected immediately post-exercise (single sprint).

Neither did fibrinogen concentration change significantly. Scientific articles discuss its decline during physical exertion (El-Sayed et al., 1999; Teległów, 2024) but do not agree on how it changes at low temperatures, where either an increase or no change has been observed (Neild et al., 1994; Teległów et al., 2024). One could assume that these two effects balance each other out, especially since relevant studies report no significant changes in this indicator (Lombardi et al., 2011; Parkkila et al., 2021). No significant differences in fibrinogen concentration were found in one of the few studies employing a methodology similar to that of this own study, in which 16 males with coronary artery disease underwent the following three tests in random order: resting while seated at a temperature of –15 °C, moderate physical effort at 22 °C, and the same physical effort at –15 °C (Parkkila et al., 2021). Feher et al. (2006), when analyzing over 6000 patients with cardiovascular diseases, found that fibrinogen levels depended primarily on the presence of disease and overall health status. It can be assumed that there is also a correlation between health status and the body response to exercise and low temperature in the context of fibrinogen concentration. The presented study did not reveal any changes in this indicator, but it is worth noting that the participants were young, healthy men. The case of Valerjan Romanovski, a Guinness World Record holder, shows an increase in fibrinogen levels of as much as 24.5% and (unlike the previously mentioned studies) a 7.5% decrease in platelet count after 50 days of exposure to extremely low temperatures in Finland. However, this may be related to a poor diet and prolonged exposure to stress caused by the cold. In addition, after such a long period of exposure, one can already expect to observe typically adaptive changes (Teległów et al., 2022).

No significant differences were observed between the experimental and control groups for any of the examined indicators in young men in blood collected immediately post-exercise (single sprint). Most likely, the low-temperature stimulation was not sufficient in young men. It is worth noting that the participants in the experimental group were wearing thermal underwear and entered the chamber after a warm-up. The study should be repeated with the use of greater cold stimulation.

The literature lacks studies combining low temperatures with physical activity. Moreover, cold stimulation is often administered by immersion in ice-cold water (Teległów et al., 2024), which, given the properties of the liquid, may affect the study results. Lombardi et al. (2011) were among the few to combine the effects of low temperature and a single bout of physical effort. In their study, 15 participants immersed in water at a temperature of 6 °C and then swam a maximum-intensity 150-meter race. The authors demonstrated an increase in platelet count and a decrease in plasma volume; however, after changing the ratio of plasma and morphotic elements to their resting values, the indicators also returned to their resting values. It was therefore hypothesized that the changes in the examined indicators resulted solely from hemoconcentration. However, in this aforementioned publication, cold stimulation also involved immersion in ice-cold water. The remaining hemorheological parameters were not assessed.

Given the discussed study results, further research is needed to examine the matter in greater detail.

### 4.1 Limitations of the study

A limitation of this study is the statistically visible baseline difference in body mass between the experimental and control groups in young men, despite the use of simple randomization. Future studies should consider stratified randomization to ensure perfect anthropometric homogeneity between groups.

## 5 Conclusions

1. It was found that a single bout of physical effort (single sprint) at low temperature affected platelet count (PLT) and plateletcrit (PCT), leading to an increase in these parameters in young men in blood collected immediately post-exercise. This is a natural effect of the release of platelet reserves (primarily from the spleen and lungs) in response to exertion, rather than a direct effect of hypothermia.
2. No significant differences were observed between the experimental and control groups for any of the examined indicators in young men in blood collected immediately post-exercise (single sprint). Most likely, the low-temperature stimulation was not sufficient in young men. It is worth noting that the participants in the experimental group were wearing thermal underwear and entered the chamber after a warm-up. The study should be repeated with the use of greater cold stimulation.

## Data Availability

The raw data supporting the conclusions of this article will be made available by the authors, without undue reservation.
